# The Brandenburg reformed medical curriculum: study locally, work locally

**DOI:** 10.3205/zma001257

**Published:** 2019-10-15

**Authors:** Andreas Winkelmann, Julia Schendzielorz, Dagmar Maske, Peter Arends, Christoph Bohne, Henrike Hölzer, Karin Harre, Jonathan Nübel, Bertram Otto, Stefanie Oess

**Affiliations:** 1 Medizinische Hochschule Brandenburg Theodor Fontane, Institut für Anatomie, Neuruppin, Germany; 2 Medizinische Hochschule Brandenburg Theodor Fontane, Prodekanat für Studium und Lehre, Referat für Curriculumsentwicklung und -koordination, Neuruppin, Germany; 3 Medizinische Hochschule Brandenburg Theodor Fontane, Prodekanat für Studium und Lehre, Referat für Studienangelegenheiten, Neuruppin, Germany; 4 Medizinische Hochschule Brandenburg Theodor Fontane, Prodekanat für Studium und Lehre, Bereich TRIK, Neuruppin, Germany; 5 Medizinische Hochschule Brandenburg Theodor Fontane, Prodekanat für Studium und Lehre, Bereich Educational Technology, Neuruppin, Germany; 6 Medizinische Hochschule Brandenburg Theodor Fontane, Prodekanat für Studium und Lehre, Bereich Simulationspatienten, Neuruppin, Germany; 7 Medizinische Hochschule Brandenburg Theodor Fontane, Lehrpraxis, Praxis für Allgemeinmedizin, Walsleben, Germany; 8 Medizinische Hochschule Brandenburg Theodor Fontane, Medizinstudent im 9. Semester, Neuruppin, Germany; 9 Medizinische Hochschule Brandenburg Theodor Fontane, Institut für Biochemie, Neuruppin, Germany

**Keywords:** curriculum/competency-based education, curriculum/interdisciplinary studies, curriculum/problem-based learning, reformed curriculum, rural medicine, communication skills, primary health care

## Abstract

The Brandenburg Medical School “Theodor Fontane” (MHB) was founded in 2014 by municipal and non-profit institutions in Bernau, Brandenburg an der Havel and Neuruppin to train more physicians for the non-metropolitan region of Brandenburg. Since the 2015 summer term, 48 medical students have been enrolled each year, accepted through the university’s own selection process in which the score on the German school-leaving exam *(Abitur)* and time spent on the waiting list play subordinate roles. Tuition fees can be partially financed through scholarship agreements with regional hospitals if the applicants commit themselves to medical specialist training *(Facharztweiterbildung)* at a particular hospital. The main places of study are Neuruppin and Brandenburg an der Havel; there is a decentralized study phase from the eighth to tenth semester of study. The Brandenburg Reformed Medical Curriculum (BMM) complies with the model clause contained in the German regulations governing the licensing of medical doctors *(ÄAppO)*. The curriculum is based on problem-based learning (PBL) and focused on competencies and consists of integrated interdisciplinary modules that combine, from the very beginning, basic sciences with clinical and theoretical medical subjects. The focus on general practice is visible in the regularly held “Practical Days” *(Praxistag)* during which second-year students and above have the opportunity to observe at participating medical practices and familiarize themselves with primary care in Brandenburg. A special focus of BMM is on the acquisition and development of communication and interpersonal skills. These are imparted through a longitudinal curriculum referred to as “Teamwork, Reflection, Interaction, Communication” (TRIK). High value is placed on critical thinking and scientific scholarship and this is reflected in an eight-week academic placement in which the students independently write a research paper. Several different teaching formats ensure that, along with learning specific subjects, sustained personal development can also take place. BMM’s decentralized study phase starting in the eighth semester represents a special part of the curriculum in which students complete their clinical training in small groups at selected cooperating hospitals in Brandenburg. This phase encompasses not only hospital placements and other local patient-centered courses, but also centralized instruction via video conferencing to assure that basic sciences and clinical theory continue to be covered. Knowledge- and performance-based semester assessments, in particular OSCEs, reinforce the practical aspects of the training. These replace the M1 state medical examination in the first study phase. The first medical students are now in their ninth semester as of April 2019, making it still too early for final evaluations. The curriculum, successfully implemented to date, already satisfies core requirements of the Master Plan 2020 for undergraduate medical education *(Masterplan Medizinstudium 2020)* with the curriculum's organization and structure, curricular content, assessment formats and student admission process. With its decentralized structure, BMM specifically addresses the social and health policy challenges facing rural regions of Brandenburg. This is the first curriculum that has taken on the improvement of healthcare in rural regions as its central aim.

## Zusammenfassung

Die Medizinische Hochschule Brandenburg Theodor Fontane (MHB) wurde 2014 von kommunalen und gemeinnützigen Trägern in Bernau, Brandenburg an der Havel und Neuruppin gegründet, um mehr Ärztinnen und Ärzte für die Non-Metropolen-Region Brandenburg auszubilden. Seit dem Sommersemester 2015 werden jährlich 48 Medizinstudierende aufgenommen, die über ein eigenes Auswahlverfahren ausgewählt werden, in dem Abiturnote und Wartezeit eine nachrangige Rolle spielen. Studiengebühren können durch Stipendienverträge mit regionalen Kliniken teilfinanziert werden, wenn sich die Bewerber/innen zur Weiterbildung in der Klinik verpflichten. Das Studium findet überwiegend an den Standorten Neuruppin und Brandenburg an der Havel statt, im 8. bis 10. Semester folgt ein dezentraler Studienabschnitt. Der Brandenburger Modellstudiengang Medizin (BMM) folgt der Modellklausel der ÄAppO. Das Curriculum ist POL-basiert und Kompetenz-orientiert und besteht aus integrierten interdisziplinären Modulen, die von Anfang an Grundlagen-, klinisch-theoretische und klinische Fächer zusammenbringen. Der Fokus auf Allgemeinmedizin schlägt sich unter anderem im regelmäßigen „Praxistag“ nieder, an dem die Studierenden ab dem 2. Semester bei niedergelassenen Lehrärztinnen und -ärzten hospitieren und über eine Integration in den Praxis-Alltag die ambulante Versorgung im Land Brandenburg kennen lernen. Ein besonderer Schwerpunkt des BMM liegt auf Erwerb und Förderung kommunikativer und sozialer Kompetenzen. Diese werden durch ein Längsschnittcurriculum „Teamarbeit, Reflexion, Interaktion, Kommunikation“ (TRIK) vermittelt. Dem wissenschaftlichen Denken und Arbeiten wird ein hoher Stellenwert beigemessen, der sich unter anderem in einem achtwöchigen Wissenschaftspraktikum niederschlägt, in dem die Studierenden eine eigenständige Forschungsarbeit erstellen. Mehrere Lehrformate stellen sicher, dass neben dem „Fach“-Unterricht eine nachhaltige Persönlichkeitsentwicklung stattfinden kann. Eine Besonderheit des BMM stellt das dezentrale Studium ab dem 8. Semester dar, in dem die Studierenden in Kleingruppen an ausgewählten kooperierenden Kliniken in Brandenburg ihre klinische Ausbildung absolvieren. Dieser Abschnitt umfasst neben Stationspraktika und weiteren lokalen Patienten-nahen Lehrangeboten auch zentralen Unterricht, der über ein Videokonferenznetzwerk realisiert wird und die weitere Beteiligung der Grundlagen- und klinisch-theoretischen Fächer sicherstellt. Wissens- und Performanz-basierte Semesterabschlussprüfungen unterstützen, insbesondere durch OSCEs, die Praxis-Orientierung der Ausbildung. Sie ersetzen im ersten Studienabschnitt die M1-Staatsprüfung. Die ersten Medizinstudierenden sind ab April 2019 im 9. Fachsemester, so dass es für abschließende Beurteilungen noch zu früh ist. Das bisher erfolgreich etablierte Curriculum erfüllt bereits heute in Bezug auf Aufbau des Studiengangs, Ausbildungsinhalte, Prüfungsformate und Studierendenauswahl zentrale Forderungen des „Masterplans Medizinstudium 2020“. Mit seiner dezentralen Struktur adressiert der BMM spezifisch die gesellschaftlichen und gesundheitspolitischen Herausforderungen der Non-Metropolen-Region Brandenburg. Er ist der erste Studiengang, der es sich zentral zur Aufgabe macht, die ärztliche Versorgung in ländlichen Regionen zu verbessern.

## 1. Introduction

Since the 2015 summer term it is possible to study medicine and psychology at the Brandenburg Medical School “Theodor Fontane” (MHB). After Witten-Herdecke, MHB is the second German university not funded by the state to offer a medical degree program as a product of its own efforts, and not as a franchise model [1]. A non-profit entity serves as the board of trustees for MHB, with four municipal and one non-profit shareholders that include the three university hospitals: the Heart Center in Bernau belonging to the Immanuel-Diakonie (including the Rüdersdorf Psychiatric Hospital), the Municipal Hospital in Brandenburg an der Havel, and the Ruppiner Hospital in Neuruppin.

The impetus behind founding MHB was the dearth of physicians in the German federal state of Brandenburg. This lack affects not only physicians who are practicing privately, but also the rural hospitals whose difficulties in recruiting qualified staff increase the farther away the hospital is located from Berlin and Potsdam. This prompted MHB’s three university hospitals to take medical education into their own hands and create their own medical school. Studying in a rural area increases the probability that graduates will remain in the region [2], [3]. Even more promising in this regard is MHB’s scholarship system: hospitals in the region assume a large part of tuition costs if the students commit to completing their specialist training at a specific hospital. Currently, around two-thirds of the medical students are taking advantage of this scholarship program.

In addition, MHB is responding to the demographic transition affecting rural regions with a particular desire to meet the needs of a society with longevity of life. To best prepare students for this, the university has chosen to focus on gerontology and health services research.

Presently, 48 medical students are enrolled each summer term (beyond this, no further focus will be placed on psychology here). The first cohort is in its ninth semester of study as of April 2019. During the admission process at MHB, the score on the German school-leaving exam *(Abitur)* and time already spent on the waiting list play subordinate roles. This elaborate admissions process is meant to select applicants who fit MHB’s curriculum, show aptitude for the medical profession, have good prospects for graduating and will remain in Brandenburg. Coming from a rural area, one of the most important predictors for later rural practice [vgl. [4]], is indirectly rated positively during the selection process. The selection process has three steps: if the formal criteria have been met, the application and statement of intent are then evaluated by faculty members. The applicants who have been rated the highest are then invited to interview on campus. This day-long event encompasses individual interviews, multiple mini-interviews, and the completion of a group assignment. Evaluations are gathered from multiple examiners using different assignments, questions and standardized assessment tools. The total number of points yields a ranked list of all applicants.

## 2. Project description: The curriculum

The Brandenburg Reformed Medical Curriculum (BMM) is based on experience with the reformed medical curriculum at Berlin’s Charité [5] and has been further developed and adapted to meet the aims of MHB and the needs of a small faculty in the process of being established. The curriculum complies with the model curriculum clause (§41 ÄAppO), as well as other requirements laid down in the medical licensing regulations and EU Directive 2005/36/EC.

**Figure 1:**
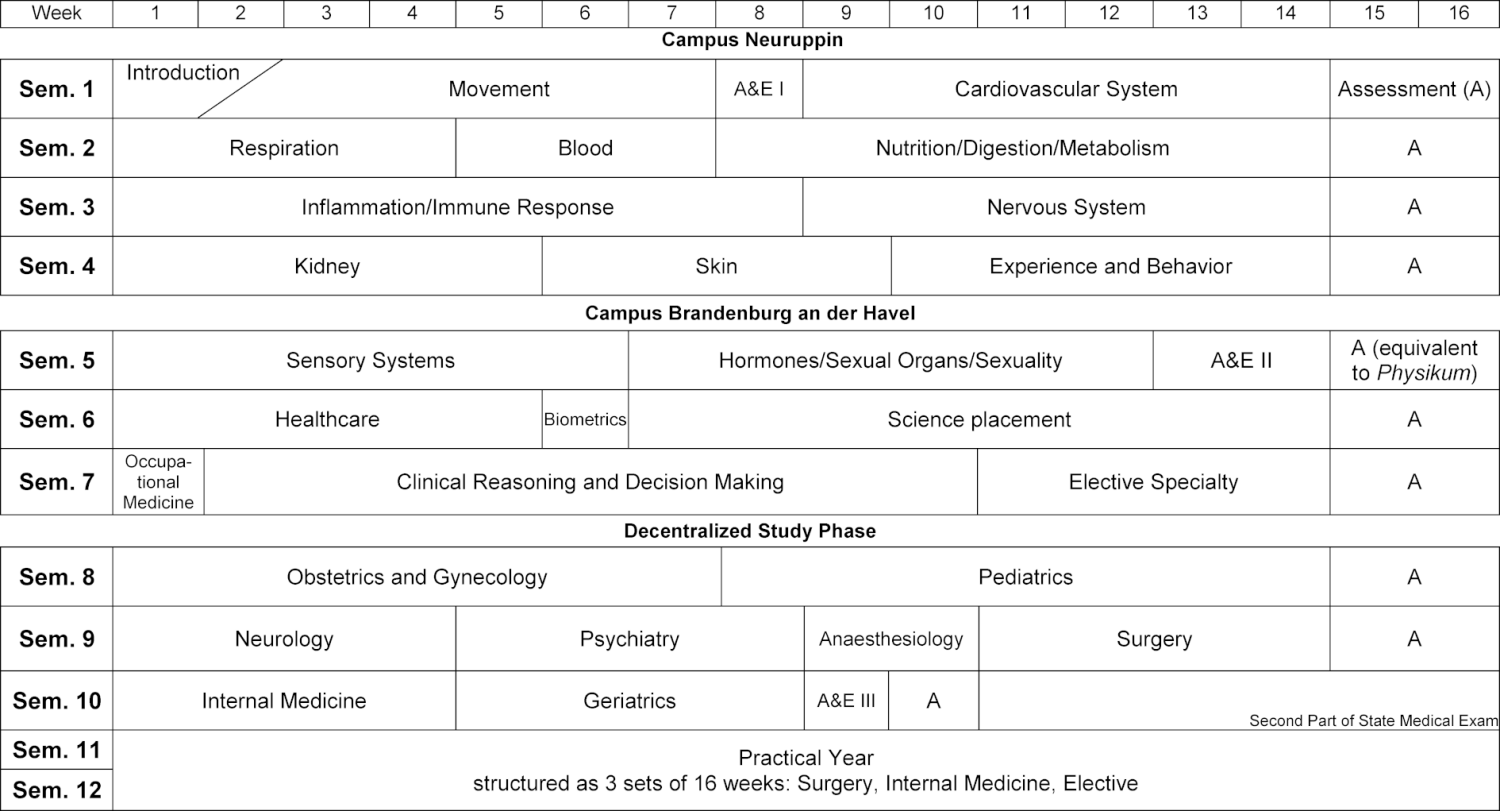
An overview of the modules in the Brandenburg Reformed Medical Curriculum

The curriculum [6] is thoroughly modular in its structure (see figure 1 (Fig. 1)). Each of the 30 modules is designed and planned by an interdisciplinary group of representatives from the subject disciplines with support from the office of the Vice-Dean for Education. The planning groups define the learning objectives that embody the requisite knowledge, skills and professional attitudes and conduct.

**Figure 2:**
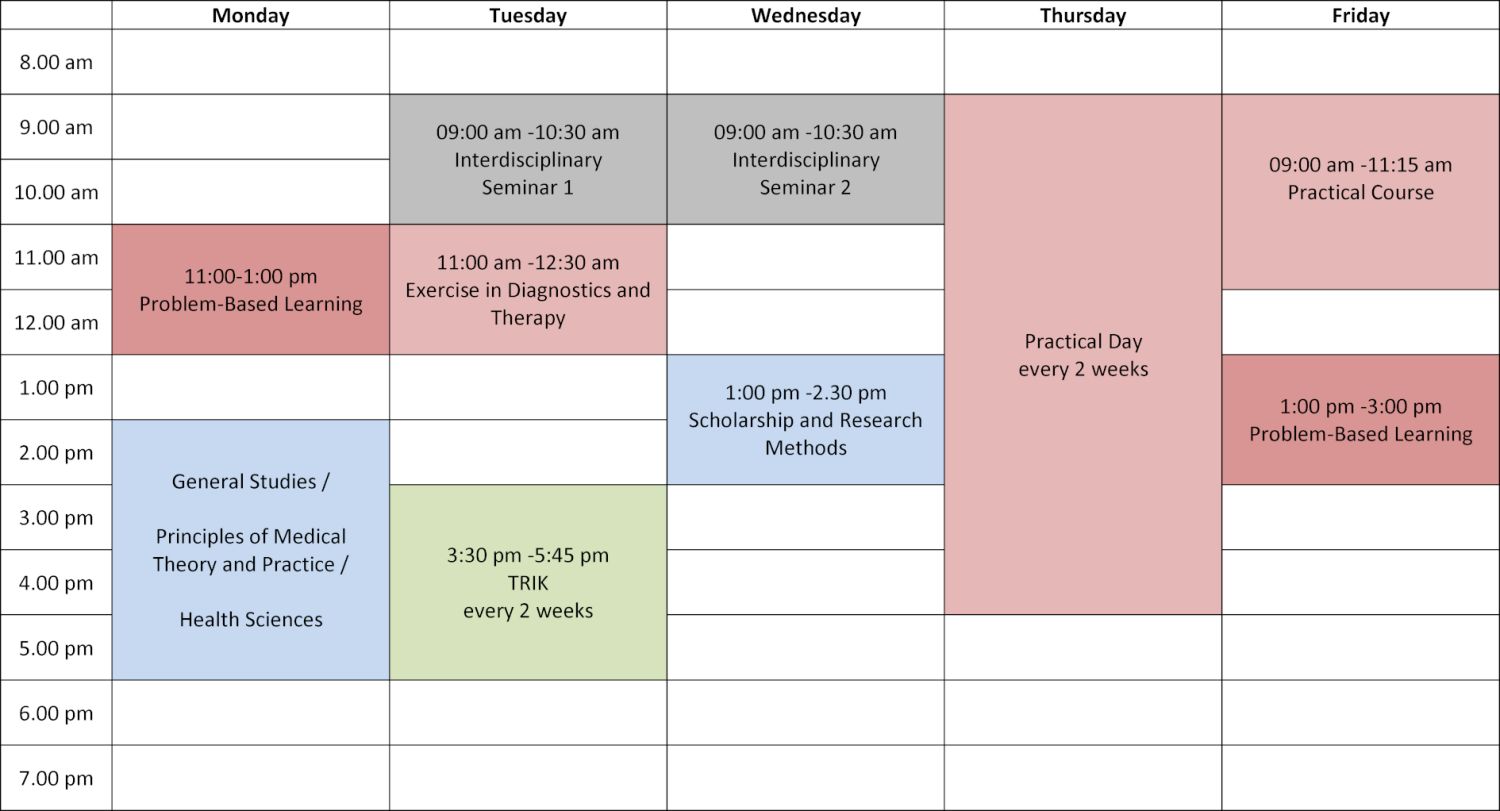
An example schedule for students taking the modules in the second to fifth semesters

The central teaching and learning format is problem-based learning (PBL). The curriculum is practice-centered, student-centered, science-based, and competency-based and focuses on interdisciplinary thinking. The program aims to teach patient-centered, personalized medicine [7] and fosters communication and interpersonal skills, along with encouraging personal development. There is no separation between the pre-clinical and clinical phases: at the beginning of the curriculum modules focusing on the basic sciences include clinical content, while at the end of the program basic sciences content is covered in clinically defined modules. There is a smooth transition, instead of having a complete split after the preliminary medical examination* (Physikum)*. A special feature of the program is its distribution over several places of study in Brandenburg (see figure 2 (Fig. 2)). The first four semesters, consisting mainly of modules dealing with the organ systems, are taught in Neuruppin. Selected courses are also offered at other places in this period, for instance at the Bernau Heart Center, the University Psychiatric Hospital in Rüdersdorf and the University Dermatology Clinic in Dessau. After moving to the campus in Brandenburg an der Havel, students take two additional organ system modules in the fifth through seventh semesters, plus an eight-week science module and modules providing a transition from general knowledge to clinical approaches. From the eighth to tenth semester there is a decentralized study phase that, above all, is comprised of clinical modules for which students are assigned in small groups to hospitals in different areas in Brandenburg and the neighboring regions (see below). Students attend courses on site and receive joint instruction via video conferencing systems. We discuss the individual teaching formats in more detail in the following.

### 2.1. PBL: from problem-based to patient-centered learning

The curriculum is student-centered, meaning that, in contrast to instructors, students are given a more active role in the learning process than is the case in traditional teaching. Problem-based learning forms the basis for students’ learning process. At the beginning of each week, groups of eight students (see figure 2 (Fig. 2)) work on a specific PBL case related to the weekly module topic. Students then work on the learning objectives connected with the module session on their own in self-directed study before they discuss and reflect on the material at the end of the week in a second module session [8].

The complexity of these PBL cases, which are usually created by the hospital physicians and centrally coordinated, increases over the course of study and the cases themselves draw not only on factual medical knowledge, but also include social, economic, psychosocial and ethical issues. For this reason, focus is first placed on acquiring basic theoretical knowledge and not primarily on solving the cases. In contrast, during the second study phase clinical management of patients is the central focus (patient-centered learning). Students are expected to make and justify tentative diagnoses by applying their previously acquired theoretical and clinical knowledge. The complex process of clinical reasoning should be applied in a targeted manner while activating the relevant domains of knowledge and decision making. To accomplish this, either two cases related in content are worked on each week, or a major symptom is examined from the perspective of two different settings (e.g. outpatient/inpatient) or medical specialties (e.g. internal medicine/surgery). The selection of the major symptoms is based on *Situations as Starting Points* [9] which represent frequent reasons why patients seek primary care consultations.

#### 2.2. Subject-based teaching formats

During a typical module week (see figure 2 (Fig. 2)), different teaching formats are employed that align with the content of the weekly topic and PBL cases. In the belief that it is not so important *how* students master the modules’ learning objectives or self-study PBL objectives, the courses that impart this knowledge and skills – as opposed to PBL – do not have mandatory attendance. Experience shows that the courses are nevertheless attended by almost all students.

The few lectures are used to provide introductions to complex topics and overviews of a topic. Medical subject knowledge is primarily taught in interdisciplinary seminars (IDS), which are designed around a specific topic by two instructors from different specialties, usually from the basic sciences and clinical subjects. This interdisciplinary approach links learning material in the different subjects (mainly anatomy, biochemistry, physiology) and the subjects in clinical theory (mainly pathology, microbiology, pharmacology) with the content of the clinical subjects.

Practical courses, in contrast, are offered in a single subject and encompass both the basic sciences (e.g. anatomy) and the clinical and theoretical clinical content (e.g. pathology, microbiology) and are designed for groups of 16 students. In the first three semesters, tutorials in the natural science are also offered to compensate for the differences among the students regarding prior education and serve to deepen their understanding of the natural sciences.

The exercises in diagnostics and therapy (EDT) primarily serve the acquisition of practical skills (e.g. physical examination, subject-specific history taking, ultrasound, bedside test) and take place in small groups of eight students. Tandem EDTs in which physicians and specially trained student tutors team teach have proven to be particularly valuable [10].

#### 2.3. Teaching and training practical skills in the BMM curriculum: the Skills Lab

The central location for teaching and training practical skills, including communication skills, is the MHB Skills Lab at both campuses in Neuruppin and Brandenburg. On the one hand, the Skills Lab is the place for curricular teaching; on the other, it provides a learning environment for self-directed study. It distinguishes itself, among other ways, by offering extra-curricular courses with a structured tutorial program in which peer-assisted learning with trained student tutors represents the central teaching and learning format.

The MHB Skills Lab was certified by the German Association for Medical Education (GMA) in March 2018 and, as a result, is the first certified Skills Lab in the German-speaking countries.

#### 2.4. Teamwork, Reflection, Interaction and Communication (TRIK)

One specific focus of the BMM curriculum is on the acquisition and encouragement of communication and interpersonal skills. This is done in line with international recommendations in education research [11] and the results of alumni surveys that show a great need for job-related communication skills [12]. These competencies are imparted in the form of “Teamwork, Reflection, Interaction and Communication” (TRIK). TRIK represents an independent, longitudinal communication curriculum that is linked with module content and taught to small groups of eight students over the course of ten semesters. This curriculum addresses many of the challenges in communication that are faced by medical professionals. The topics of the individual semesters build on each other and are sequenced in such a manner that they become increasingly complex and more demanding over the course of the program, much like learning spirals. This ranges, for instance, from recording case histories and holding patient consultations on prevention and health promotion to applying the concept of shared decision-making [13]. A weekend workshop in the eighth semester is dedicated to interprofessional communication.

As with the practical assessments, the teaching format for TRIK makes use of simulated patients, meaning that lay actors take the role of patients [14]. This enables students to practice using their current knowledge in appropriate role play exercises without importuning real patients. Presently, around 40 lay actors have been recruited for both campus locations. The simulated patients provide feedback to students on how they experienced the doctor/patient interaction and give students the opportunity to reflect on how their attitudes and conduct affect patients.

#### 2.5. Practice Days

Practice Days *(Praxistage)* ensure the practical orientation of the curriculum from the very beginning and familiarize students with the realities of outpatient care in Brandenburg with a focus on general practice. At intervals of 14 days in the second to fifth semesters, students observe at medical practices in the surrounding area and are integrated into the daily routines. These medical practices belong to general practitioners; however, in the fourth and fifth semesters other specialists are also included. Along with practical application of the acquired skills in taking medical histories and physical examination techniques, the most important aspect of the Practice Days involves enabling students to gain insights into primary care. Students are intentionally confronted with what it means to provide long-term care, prevention and staged therapy in the outpatient setting; furthermore, they are given opportunities to collaborate with other healthcare professions. There is currently a network of 119 medical practices whose physicians are included as MHB faculty members. The Association of Statutory Health Insurance Physicians in Brandenburg (KVBB) supports this project and has made university-level teaching a component of its certification program.

#### 2.6. Longitudinal curriculum focusing on scholarship

Critical thinking and scholarship are promoted in a special longitudinal curriculum that consists of a seminar series, a one-week-long module on Biometrics and an eight-week-long academic placement. The core assignment is an individual research paper as part of the academic placement in the sixth semester. The research issues are primarily of an experimental, clinical and epidemiological nature and conceived of so that the research project can be reasonably completed within the time allotted. Academic achievement and learning is demonstrated in the form of a scientific paper, at least 12 pages in length, and a poster which is then presented by the student at a university conference at the end of the semester and evaluated by an expert committee. The first time this was done in the 2017/18 winter term, the majority of topics were in clinical research (43%), followed by healthcare research (26%), basic sciences research (21%) and educational research (10%).

#### 2.7. Longitudinal courses independent of the modules

Under the heading of Human and Health Sciences, multiple teaching formats ensure that, along with instruction on the module subjects, sustained personal development can take place so that graduates can assume a professional attitude, reflect on how they practice medicine, and accurately assess their own competencies. Belonging to these particular courses is a second-semester seminar series on health sciences that covers not only general issues involving healthcare, but also specific challenges to providing healthcare in Brandenburg. Among other things, students receive an initial overview of the German healthcare system before they deal with topics such as the economy and the healthcare structures for palliative medicine, hospice and psychiatric care. Delving even deeper into these issues takes place in the sixth-semester module on healthcare.

Second, there are the seminars on the "principles of medical theory and practice" in the third, fourth and eighth semesters, as well as the “general studies” courses in the second to sixth semesters which cover humanities perspectives on medicine and non-medical topics [see [15]. The aim is to initiate interdisciplinary reflection to encourage various critical thinking and decision-making concepts that should be used in later practice. Students are able to choose between courses in art, history and ethics (e.g. medicine during the Nazi era), philosophy (e.g. philosophical and psychoanalytical approaches) and sociology (e.g. medicine and gender).

In addition, students complete placements and participate in job shadowing to learn about specific professional fields (as laid down in the *ÄAppO*) between their first and fifth semesters. Students either organize these themselves according to defined criteria and their own interests, or they take part in opportunities organized by the university (e.g. medical services offered by the statutory health insurance providers, community service in connection with Doctors without Borders, or medical care in penitentiaries).

#### 2.8. Decentralized Study

**Figure 3:**
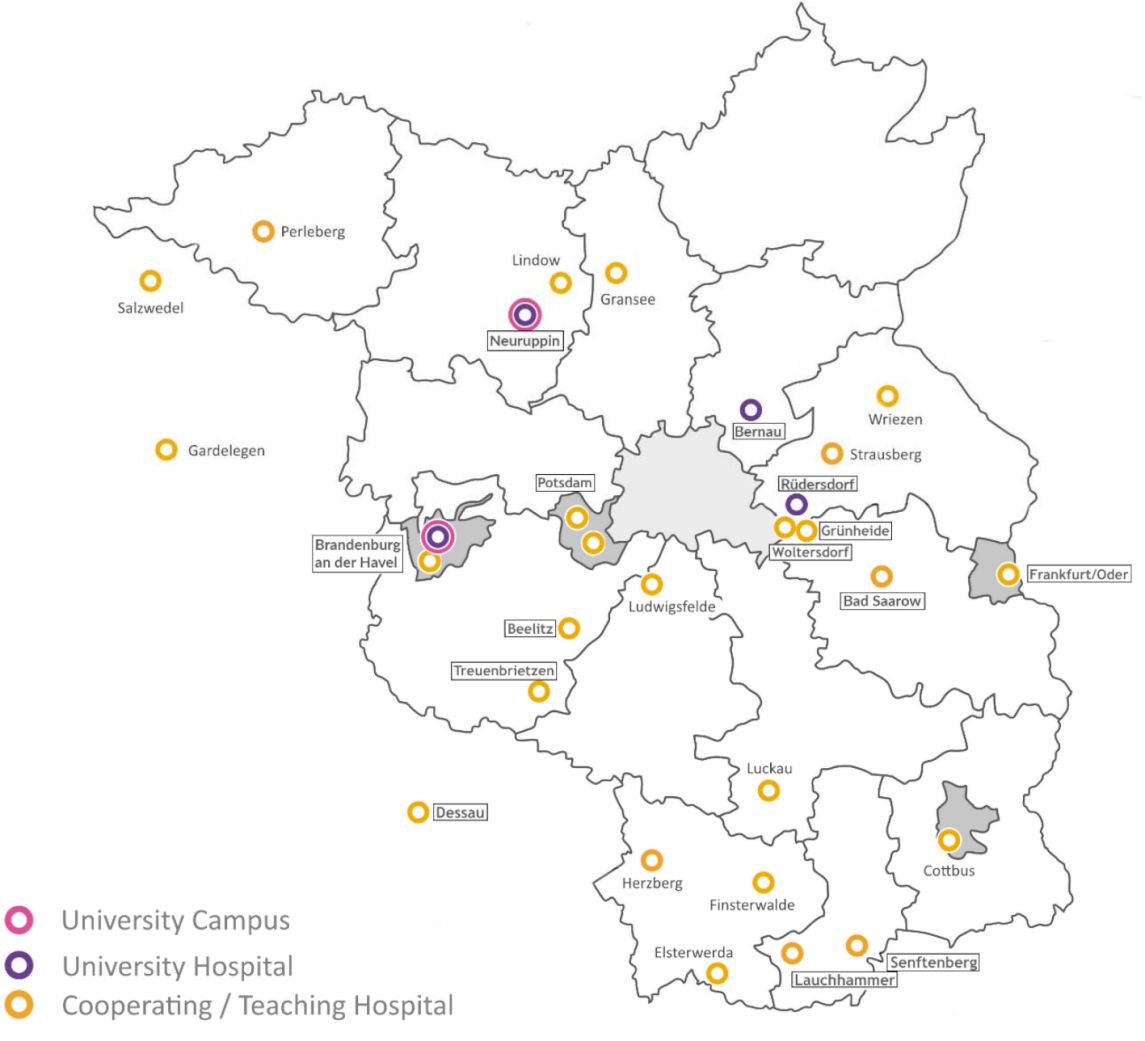
The hospitals affiliated with MHB in the German state of Brandenburg in March 2019. The names of the hospitals participating in the decentralized study phase appear in boxes. The locations listed participate in teaching and/or provide scholarship agreements to students.

During the decentralized study phase in the eighth to tenth semesters, students continue their studies in small groups at one of seven regional “anchor locations.” Such an anchor location includes up to five rural university hospitals, academic teaching hospitals or cooperating hospitals which, between them, offer all of the clinical subjects in the decentralized study phase (see figure 1 (Fig. 1)). In total, around 20 highly committed hospitals from across Brandenburg and the neighboring regions have been chosen to cooperate in this reformed curriculum (see figure 3 (Fig. 3)). The core teaching and learning format for patient-centered training is the mandatory hospital placement which focuses on clinical differential diagnostics in the inpatient setting. In addition to PBL, both TRIK and bedside teaching are offered at the hospitals; weekly case conferences are held during which students study clinical cases and their progression and then present them in their small groups. The decentralized program is supplemented by lectures and IDS.

The lectures and IDS have been implemented as central courses using an intelligent video conference network that provides stable transmission between specially equipped video conference rooms. These rooms have multiple monitors, microphones and cameras with speaker tracking. This network enables interaction that is very much comparable to courses that are held in person.

#### 2.9. Assessments

Assessments are administered at the end of each semester and consist of written and practical components. These are governed by rules and regulations approved of by the state ministry and are based on the learning objectives that have been defined by the particular planning committees for the modules.

The written assessments consist of semester-relevant multiple-choice (MC) exams. The questions have been drafted in a review process. Each test has been compiled in cooperation between the assessment office and those responsible for the module.

Up until the seventh semester, the practical assessments take the form of semester-relevant OSCEs (Objective Structured Clinical Examination) with eight to ten stations at which practical and communication skills are evaluated, in part using simulated patients. These stations are designed by the assessment office in cooperation with the teachers at the hospitals. One to two OSCE stations per semester assessment can also take place as OSPEs (Objective Structured Practical Examination) in which the basic science subjects are tested, for example, as an anatomy tag test [16]. During the decentralized study phase, a workplace-based clinical assessment is administered in combination with a structured oral assessment based on the testing modes in the third section of the state medical examination.

In compliance with the university rules and regulations governing assessments and the medical licensing regulations, students obtain the equivalent of the preliminary medical examination *(Physikum)* after passing all of the assessments in the first through fifth semesters. The second and third sections of the state medical examination are administered in compliance with the medical licensing regulations.

## 3. Results

A final evaluation of the BMM curriculum will only be possible after multiple cohorts have completed the entire program; an effect on medical care in the region will only become perceptible after many years. Hence, we can only point to some positive developments and stumbling blocks here.

The BMM curriculum is faced with the general challenges of a reformed curriculum. Many instructors who are only familiar with the traditional mode of study must be convinced of the merits of the new approach. According to the authors’ experience, those representing the medical disciplines must often become accustomed to the idea that they can no longer teach the entire curriculum from the front of the classroom, but rather must trust that the students can learn on their own through PBL and self-guided study. In our experience, faculty development also has to include convincing medical teachers that only a reduction in teaching material, for instance, by learning through example, can do justice to the ever increasing body of medical knowledge and also make room for achieving other study goals. The centralization of curriculum planning and coordination typical of reformed curricula and the unfamiliar confrontation with “education experts” from outside the taught discipline sometimes lead to feelings of a restricted “academic freedom.” However, central module planning and constant teacher evaluations are necessary to ensure that standards are met, especially given the geographical distribution of teachers across many locations. MHB has established diverse teacher trainings to familiarize instructors with teaching formats such as PBL, TRIK, interdisciplinary seminars and online teaching. Overall, since its founding MHB has trained 459 physicians, of which 211 received PBL teacher training. Medical teacher training for 156 participants has already taken place at the decentralized study locations.

One challenge specific to MHB is the distribution of the curriculum over multiple locations which are all more than one hour’s drive from each other and are not easy to reach using public transportation. Students must accept that moving several times will simply be a part of studying medicine, while instructors often have long journeys to the places where they teach. For some courses, the medical school organizes transportation for students (e.g. to the anatomical dissecting room that is only located in Neuruppin or for specific subjects in Bernau, Rüdersdorf, Dessau or Senftenberg; see above).

Another challenge is the establishment of an entirely new medical school including the acquisition and equipping of rooms and the organization of infrastructure (Skills Lab, university library, IT service, canteen, student centers).

In general, the curriculum has been successfully implemented. The model curriculum is viewed positively overall and MHB has seen a very low drop-out rate to date in comparison with state-run universities [17] (currently under 5% for the first cohort) and unceasingly high numbers of applicants. The personal atmosphere arising from the low numbers of students has a positive effect. All of the modules and teaching formats that make up the BMM curriculum are subject to continual evaluation by a centrally organized evaluation office and the evaluation results are systematically used for further development of the curriculum’s organization and curricular content. The extensive student evaluations have approximately 15 items for each module and ask about the time used for self-directed study, the degree to which the curriculum is over-challenging or under-challenging, students’ subjective learning success, satisfaction with the program’s organization and enjoyability, as well as an overall evaluation. In addition to this, each course is rated using a 7-point Likert scale and open-ended comments can be submitted. The evaluation results are taken into account in the module planning for the following year and communicated to the affected teachers.

Overall, the modules have been positively rated on average. As an example, overall satisfaction with the six-week-long module on hormones, sexual organs and sexuality that is offered in the fifth semester was 81% and 87% for the first two times that it has been offered. Student comments in the 2017 summer semester pointed out an unequal distribution of the learning material within the module with partly over-challenging and partly under-challenging module weeks and PBL cases that were too similar. This was taken into account in the planning for 2018 by redistributing the content over the module weeks and exchanging PBL cases. This was acknowledged in the evaluation of the most recent module cycle for which the corresponding items, “I am satisfied with the organizational coordination of the courses” and “I am satisfied with the alignment of the course content with the weekly PBL topic,” increased from 4.1 (MV, SD 1.38) and 3.8 (MV, SD 1.85) to 5.1 (MV, SD 1.56) and 5.6 (MV, SD 1.1), respectively. Responding rapidly to evaluation results has led to positive student perceptions of the MHB curriculum. And finally, students clearly emphasized in their YouTube video in the 2018 “Medi” championships that “our curriculum is the cream of the crop!” [18].

Naturally, there are no state medical examination results available yet to compare with other medical schools. However, first results on the progress test [19], in which MHB students participate each year, show knowledge gains and proficiency growth comparable to other reformed curricula. Up to and including the fifth semester, the first two cohorts are at or above the average for all participating medical schools.

## 4. Discussion and Conclusion

For some time now efforts have been made to reform medical education in Germany not only in response to the demographic shifts, scientific discoveries in the life sciences and the resulting innovations in diagnostics and therapies, but also to the digitalization of medicine and the treatment of patients who have full access to online information. It was within this context that the German Council of Science and Humanities (*Wissenschaftsrat)* gave its recommendations in 2014 on further development of the medical curriculum [20]; this was done using existing reformed curricula. The Master Plan 2020 for undergraduate medical education put forth by the German Federal Ministry for Education and Research was drafted in 2017 incorporating suggestions from the national and state working group along with other professional associations and student bodies [21].

This master plan provides for reforms, in particular for increasing relevancy to practice, improving the links between preclinical and clinical curricular content, and strengthening general practice and competencies revolving around communication, interpersonal skills and academic scholarship. All of this strives for medical education that is patient-centered, interdisciplinary and interprofessional. With the Brandenburg Reformed Medical Curriculum, we have already implemented the required measures to a large extent and are educating our medical students according to the reforms that have been called for.

The special needs for healthcare in rural Brandenburg are being specifically addressed by both the early inclusion of practicing physicians as part of the Practical Days held during the second to fifth semesters and the design of the curriculum for the second study phase that involves a network of cooperating hospitals. As a result, students are able to experience different potential workplaces in Brandenburg beyond the campus setting, which is meant to have a positive effect in that students will choose to remain in the area and go on to practice professionally. Similar “sticking effects” have already been described in alumni surveys [3], [4]. These efforts are supported by the personal atmosphere that exists between teachers and students and the close contact between students and practicing clinicians who over the course of the program become role models and mentors. Since the fields of gerontology and health services research are the main research focus at MHB, Brandenburg’s larger concerns as an area affected by the demographic shift are also reflected in the academic placement and the related science curriculum. Thus, the BMM is in our opinion the first curriculum in Germany that has made improving medical care in rural regions its central aim. How well we meet this objective is something by which we will be measured in the long term.

## Competing interests

The authors declare that they have no competing interests.
